# Characteristic distribution and molecular properties of normal cellular prion protein in human endocrine and exocrine tissues

**DOI:** 10.1038/s41598-022-19632-4

**Published:** 2022-09-10

**Authors:** Sachiko Koyama, Hideko Noguchi, Kaoru Yagita, Hideomi Hamasaki, Masahiro Shijo, Motoi Yoshimura, Kohei Inoshita, Naokazu Sasagasako, Hiroyuki Honda

**Affiliations:** 1 Department of Neuropathology, Graduate School of Medical Sciences, Kyushu University, 3-1-1 Maidashi, Higashi-ku, Fukuoka, 812-8582 Japan; 2 Division of Neurology, Department of Neurology, Neuro Muscular Center, National Omuta Hospital, Fukuoka, Japan

**Keywords:** Central nervous system infections, Prion diseases

## Abstract

Prion disease is an infectious and fatal neurodegenerative disease. Human prion disease autopsy studies have revealed abnormal prion protein (PrP^Sc^) deposits in the central nervous system and systemic organs. In deer, chronic wasting disease has also become a global problem, with PrP^Sc^ in saliva and feces. Therefore, understanding normal cellular prion proteins (PrP^c^) characteristics in human systemic organs is important since they could be a PrP^Sc^ source. This study used western blotting and immunohistochemistry to investigate endocrine and exocrine tissues, such as the human pituitary, adrenal, submandibular glands and the pancreas. All tissues had 30–40 kDa PrP signals, which is a slightly higher molecular weight than normal brain tissue. Most cytoplasmic PrP-positive adenohypophyseal cells were immunopositive for nuclear pituitary-specific positive transcription factor 1. The adrenal medulla and islet cells of the pancreas were PrP-positive and colocalized with chromogranin A. The duct epithelium in the submandibular gland and pancreas were immunopositive for PrP. This study reports the characteristic molecular properties and detailed tissue localization of PrP^c^ in endocrine and exocrine tissues, which is important for infection control and diagnosis.

## Introduction

Human prion diseases, also known as transmissible spongiform encephalopathies, are lethal neurodegenerative disorders, including sporadic Creutzfeldt-Jakob disease (sCJD), inherited prion disease, and acquired human prion disease. Acquired human prion diseases include iatrogenic CJD (iCJD), kuru, and variant CJD. Notably, among the iCJD cases, many received human growth hormone (GH) derived from cadaveric pituitaries^1^.

In some prion diseases, abnormal PrP deposits have been identified in systemic organs other than the central nervous system^2–4^. Prion disease is characterized by the conversion of a normal cellular prion protein isoform (PrP^c^) into an abnormal pathogenic PrP (scrapie prion protein: PrP^Sc^). PrP^Sc^ has high β-sheet content and is resistant to proteinase K treatment (PrP^res^). Therefore, it is important to clarify the histological localization of PrP^c^ in systemic organs, which can be a source of PrP^Sc^. Previously, we demonstrated that cytoplasm from anterior pituitary gland adenohypophyseal cells is rich in PrP^c^ and that the pituitary gland of patients with prion diseases contains PrP^res^^5^.

There are already many reports on PrP^c^ localized in systemic organs in animals. In particular, PrP is related to endocrine tissues (e.g., the pituitary and adrenal glands and the pancreas) and endocrine pathway crinophagy^6–10^. In addition, chronic wasting disease (CWD), a prion disease of deer and elk, has become an issue in the United States, Canada, and Europe^11,12^. Transmission routes are thought to be prion-contaminated environments derived from saliva^13,14^, urine^15^, and feces^16^.

In humans, information about the relationship between prion proteins and endocrine and exocrine organs remains limited, even regarding their histological localization. Therefore, this study clarified the histological localization and biochemical properties of prion proteins in endocrine and exocrine organs in autopsy patients with non-prion diseases.

## Materials and methods

### Subjects

We obtained pancreatic and pituitary, adrenal, and submandibular gland tissues from seven autopsy cases of non-prion diseases (one amyotrophic lateral sclerosis case, one spinocerebellar ataxia type 6 case, two multiple sclerosis cases, one myotonic dystrophy case, one Becker muscular dystrophy case, one no neuromuscular disease case: chronic liver cirrhosis) at Kyushu University Hospital, Fukuoka, Japan (Table 1). We obtained informed written consent for autopsies from all patients or their next of kin. All analyses were performed following the Declaration of Helsinki. The Ethics Committee of the Faculty of Medicine of Kyushu University (#2019-179) approved this study.

**Table 1:** Case profiles.

Case No	Diagnosis	Age at death (y)	Sex	Brain weight (g)	PMI (h)
1	ALS	79	F	1300	26
2	SCA6	93	M	1161	24
3	MS	73	M	1192	21
4	MS	43	F	980	13
5	MD	63	F	1038	4
6	BMD	46	M	1386	2
7	LC	79	M	1350	5.5

ALS, Amyotrophic lateral sclerosis; BMD, Becker muscular dystrophy; F, female; LC, liver cirrhosis; M, male; MD, myotonic dystrophy; MS, multiple sclerosis; PMI, postmortem interval; SCA6, spinocerebellar ataxia 6.

### Molecular characterization of PrP

The frontal cortex, pancreatic, and pituitary, adrenal and submandibular gland samples were obtained from formalin-fixed, paraffin-embedded (FFPE) specimens. The detailed protein extraction methods have been previously described^17,18^. Briefly, each FFPE specimen was sectioned at 10 µm to produce 10 serial sections. Next, 0.5 mL of mineral oil (Sigma-Aldrich, USA) was added to the sectioned FFPE samples and then incubated at 95 °C for 2 min to dissolve the wax. Finally, the supernatants were removed by centrifugation at 11,000×*g* for 3 min at room temperature. The incubation and centrifugation steps were repeated by adding 0.1 mL of mineral oil. Then, the pellets were resuspended in 80 µL of citrate-sodium dodecyl sulfate (SDS) buffer (200 mM Tris–HCl (pH 7.5), 200 mM NaCl, 5% SDS, and 100 mM sodium citrate), and the supernatant was removed after centrifugation at 11,000×*g* for 3 min at room temperature. Next, proteins were extracted from the pellet by incubation at 100 °C for 20 min and then at 80 °C for 2 h in citrate-SDS buffer. Finally, the supernatant was recovered after centrifugation at 11,000×*g* for 15 min at room temperature. To concentrate the protein, acetone was added to the extracted protein and incubated overnight at − 20 °C. Then, the supernatant was discarded after centrifugation at 10,000×*g* for 15 min at room temperature. Finally, the pellets were left at room temperature for 30 min to volatilize acetone, then re-dissolved in citrate-SDS buffer to prepare the samples for western blotting. Prior to western blotting, the protein concentration of the sample was adjusted. In addition, we performed deglycosylation treatment with PNGaseF (New England Biolabs, Ipswich, MA).

The frontal cortex, pancreatic, adrenal and submandibular glands samples were stored at − 80 ℃ until use. Samples were homogenized to a final concentration of 10% in lysis buffer (50 mM Tris–HCl, 150 mM NaCl, 1% Nonidet P-40, 0.5% sodium deoxycholate, and 0.1% SDS; pH 7.6). Frozen samples were limited (pancreas: case 5, adrenal gland: case 3, submandibular gland: case 1 to 5), because, in Japan, we do not collect frozen samples in routine autopsy.

**Figure 1:**
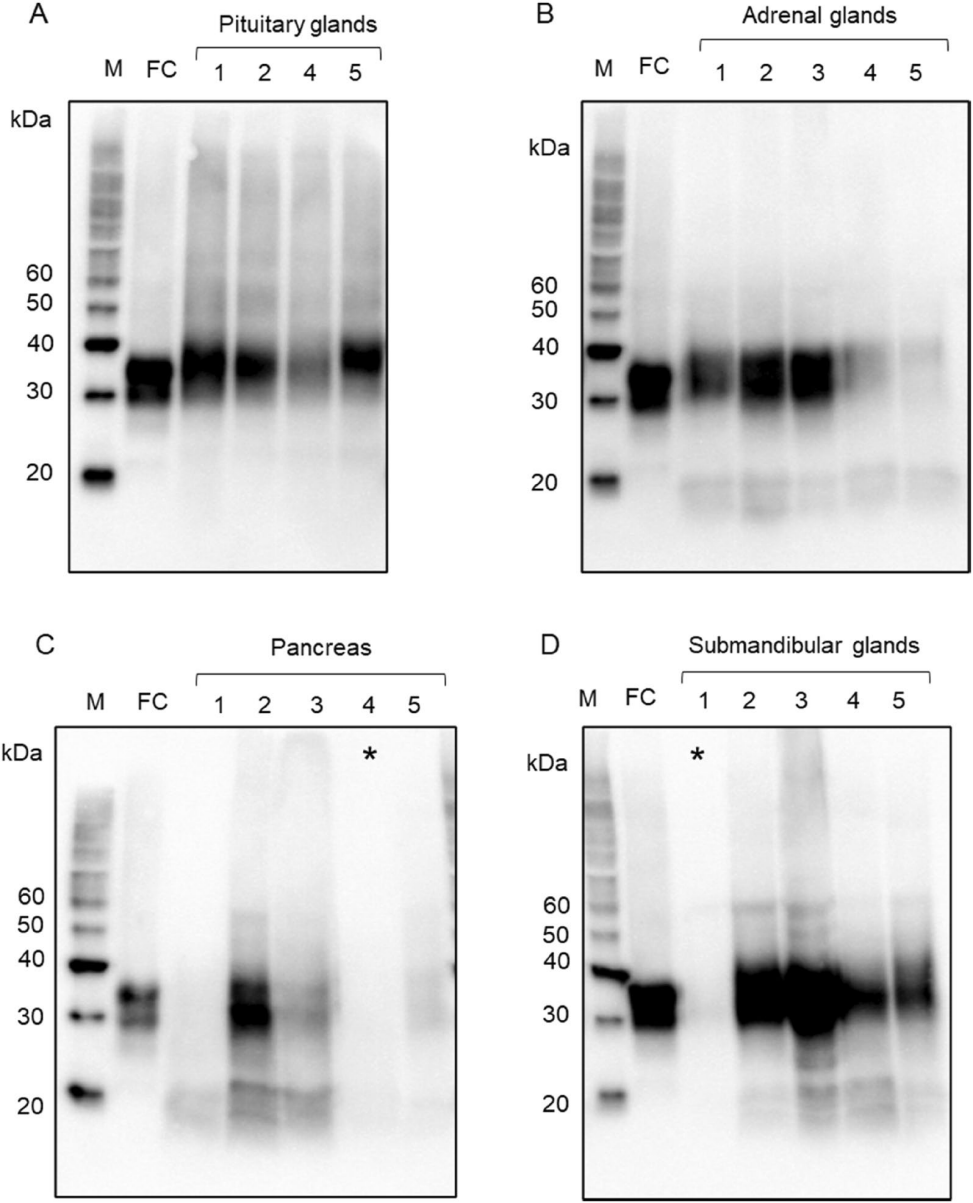
Western blot analyses from FFPE samples with the 3F4 anti-prion protein (PrP) antibody. (**A**) The frontal cortex (FC) samples show ~ 25–38 kDa PrP signals, and the pituitary gland samples (cases 1, 2, 4, and 5) show ~ 30–40 kDa PrP signals. (**B**) The adrenal gland samples (cases 1–5) show ~ 30–40 kDa and ~ 16–20 kDa PrP signals. (**C**) The pancreas samples (cases 1–5) show ~ 30–40 kDa and ~ 16–20 kDa PrP signals. The PrP signal is difficult to identify in case 4. (**D**) The submandibular glands (cases 1–5) show ~ 30–40 kDa and ~ 16–20 kDa PrP signals. The PrP signal is difficult to identify in case 1.

PrP^c^ was detected by SDS polyacrylamide gel electrophoresis in pre-made gels (12% gel; Bio-Rad, Hercules, CA) and then transferred onto polyvinylidene difluoride membranes. PrP^c^ was detected using two anti-PrP antibodies (mouse monoclonal 3F4 [specific for PrP at 109–112], 1:2000; BioLegend, San Diego, CA and rabbit monoclonal EP1802Y [specific for PrP at 212–230], 1:2000; Abcam, Cambridge, UK). Peroxidase-conjugated anti-mouse immunoglobulin (Ig) G (PI-2000, 1:20,000, VECTOR, Burlingame, CA, USA) and anti-rabbit IgG (PI-1000, 1:20,000, VECTOR) were used as secondary antibodies. Immunoreactivity was visualized using Immobilon Western Chemilum HRP substrate (Millipore, Billerica, MA, USA). After confirming the immunoreactivity with the 3F4 antibody, the primary antibody was removed using stripping buffer (62.5 mM Tris–HCl, pH 6.8, 2% SDS, 100 mM β-mercaptoethanol). Then, PrP^c^ was identified again using another PrP antibody (EP1802Y). The edges of the membranes are not visible in Suppl. Figs. S3A–D and S4, but Fig. 1, Suppl Figs. S3, S4 and S5 are the same membrane, and the crop has never been performed.

### Immunohistochemistry

Hematoxylin and eosin (HE) staining was performed for histological examination. Immunohistochemistry was performed using primary antibodies specific to anti-prion proteins: (mouse monoclonal 3F4 [1:400], mouse monoclonal 8G8 [specific for PrP at 95–110, 1:400]; Cayman, Ann Arbor, MI, USA, and rabbit monoclonal EP1802Y [1:1000]), mouse monoclonal anti-pituitary-specific positive transcription factor 1 (Pit-1; 1:100, Santa Cruz Biotechnology), rabbit polyclonal anti-T-Box Transcription Factor 19 (TBX19; 1:200, Atlas Antibodies, Bromma, Sweden), mouse monoclonal anti-steroidogenic factor 1 (SF-1; 1:100, Perseus Proteomics, Tokyo, Japan), and mouse monoclonal anti-chromogranin A (CgA; 1:100, Millipore). Double immunofluorescence staining was performed using a combination of mouse monoclonal Pit-1 and rabbit monoclonal EP1802Y antibodies or rabbit polyclonal TBX19 and mouse monoclonal 3F4 antibodies. Alexa 488-labeled anti-mouse IgG (Invitrogen, Carlsbad, CA, USA), Alexa 546-labeled anti-mouse IgG (Invitrogen), Alexa 488-labeled anti-rabbit IgG (Invitrogen), and Alexa 546-labeled anti-rabbit IgG (Invitrogen) were used as secondary antibodies. The specimens were visualized using a Nikon A1R-A1 confocal microscope (Nikon, Tokyo, Japan).

## Results

### Molecular characterization of PrP

**Figure 2:**
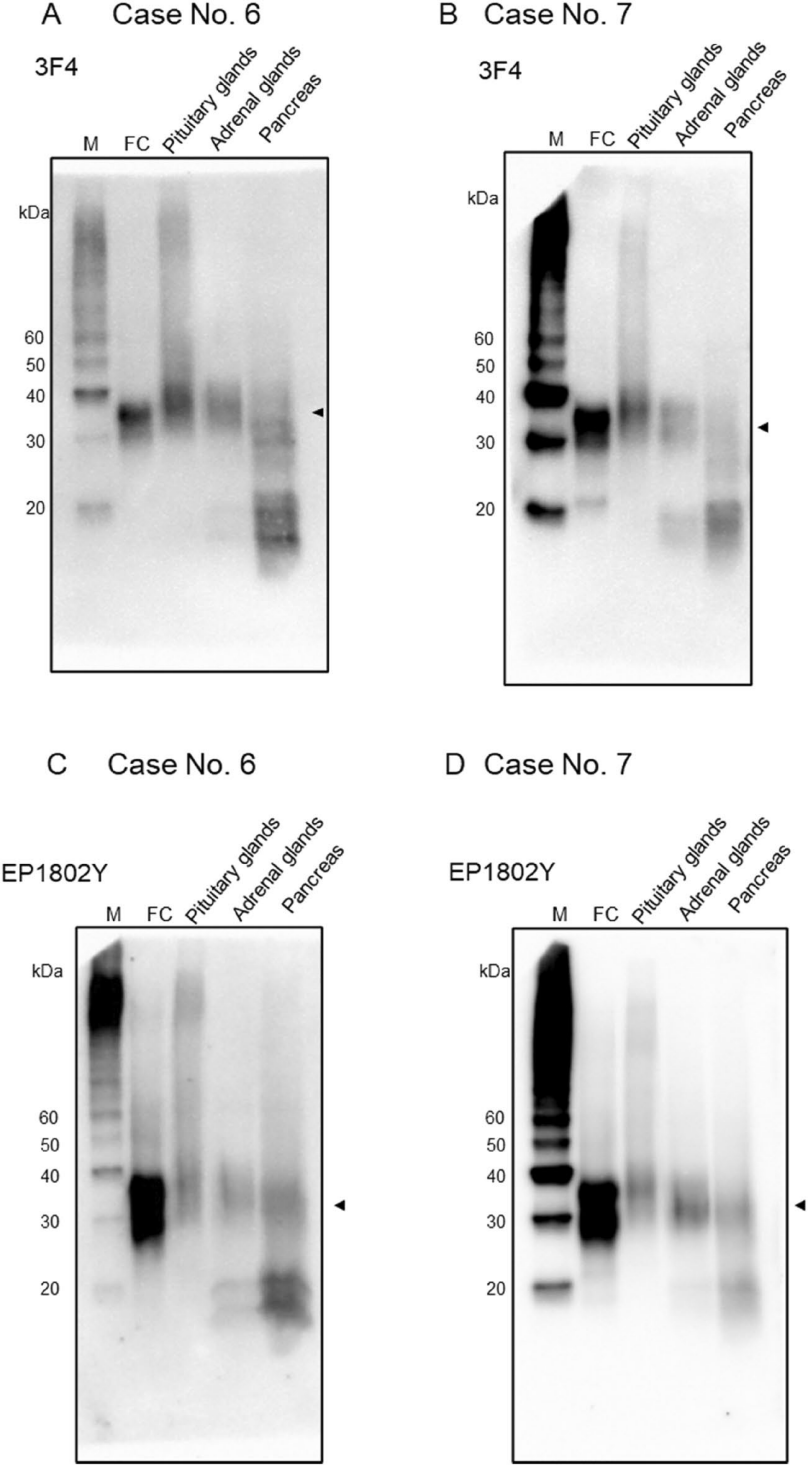
Western blot analyses from FFPE samples with the 3F4 (**A**, **B**) and EP1802Y (**C**, **D**) anti-prion protein (PrP) antibodies (case 6 and 7). (**A**) Case 6 (**B**) Case 7: Western blot analysis using 3F4, the frontal cortex (FC) samples show ~ 25–38 kDa PrP signals. The pituitary glands, adrenal glands and pancreas show ~ 30–40 kDa PrP signals (arrowhead). (**C**) Case 6 (**D**) Case 7: Western blot analysis using EP1802Y, the frontal cortex (FC) samples show ~ 25–38 kDa PrP signals. The pituitary glands, adrenal glands and pancreas show ~ 30–40 kDa PrP signals (arrowhead).

**Figure 3:**
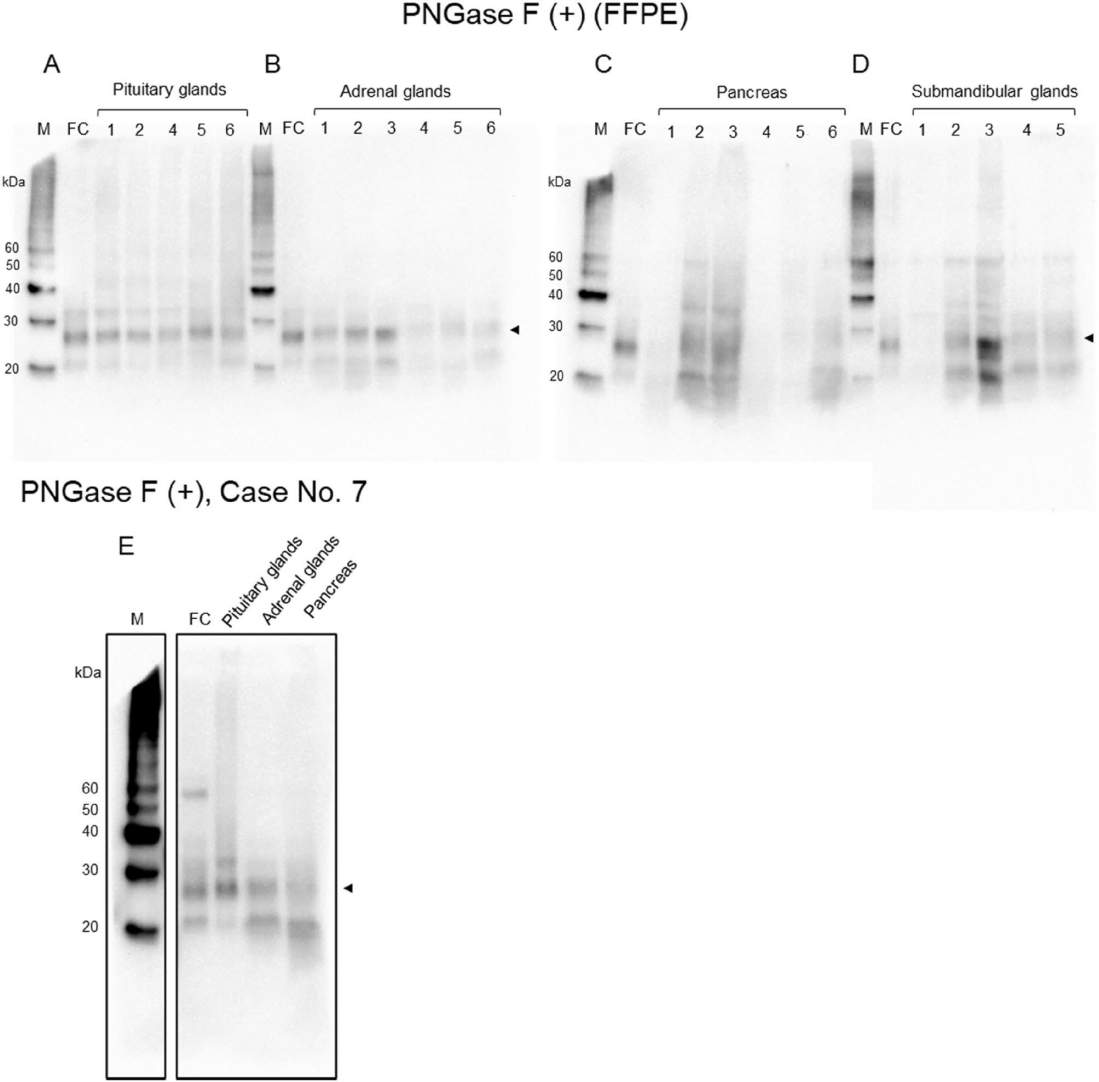
Western blot analyses from FFPE samples after deglycosylated treatment. (**A**) Pituitary glands, (**B**) adrenal glands: PrP signals are mainly observed at the molecular weight of 25 kDa (arrow head), which is correspond to nonglycosylated PrP. (**C**) Pancreas: In the case 2 and 3, PrP signals are mainly observed at the molecular weight of 25 kDa, however in the case 1, 4, 5, and 6, 25 kDa signals were not evident. (**D**) Submandibular glands: PrP signals are mainly observed at the molecular weight of 25 kDa (arrow head). However, in the case 1, PrP signals were not evident. (**E**) Case 7: In the frontal cortex, pituitary gland, adrenal gland and pancreas, PrP signals are mainly observed at the molecular weight of 25 kDa (arrow head), which is correspond to nonglycosylated PrP.

The frontal cortex samples had unglycosylated, monoglycosylated, and diglycosylated PrP signals, ranging from 25 to 38 kDa using the 3F4 antibody (Fig. 1A). The PrP signal intensity varied per tissue depending on the PrP antibody and the case (Table 2). The pituitary samples had a PrP smear band around 30–40 kDa (Fig. 1A), and the adrenal gland samples had PrP smear bands around 16–20 kDa in addition to those around 30–40 kDa. The pancreatic PrP signal pattern was nearly identical to the adrenal gland pattern, with ~ 16–20 kDa and ~ 30–40 kDa smear bands (Fig. 1C). However, the signal intensity differed between cases, and case 4 was challenging to detect (Fig. 1C, asterisk). The submandibular gland samples had PrP signal patterns similar to those of the adrenal gland and pancreas with PrP signals of ~ 16–20 kDa and ~ 30–40 kDa (Fig. 1D). However, it was difficult to detect the PrP signal in case 1 (Fig. 1D, asterisk). In the case 6 and 7, western blot analysis with 3F4 antibody and EP1802Y antibody showed PrP signals almost similar to the case 1–5 (Fig. 2). The raw image of Figs. 1 and 2 are shown in the supplemental Figs. S1 and S2 respectively. After deglycosylation treatment, almost all samples showed a prominent signal at the molecular weight of 25 kDa, which corresponds to the unglycosylated PrP molecular weight (Fig. 3). Full length membrane of Fig. 3E is shown in Supplemental Fig. S6.

**Table 2:** The molecular characteristics of prion proteins by western blot from FFPE samples with 3F4 and EP1802Y antibodies.

Case No		Pituitary glands		Adrenal glands		Pancreas		Submandibular glands	
		3F4	EP1802Y	3F4	EP1802Y	3F4	EP1802Y	3F4	EP1802Y
1	30–40 kDa	+	+	+	+	+	+	±	−
2	30–40 kDa	+	+	+	+	+	+	+	+
3	30–40 kDa	n.a	n.a	+	+	+	+	+	+
4	30–40 kDa	+	+	+	+	−	+	+	+
5	30–40 kDa	+	+	+	+	+	+	+	+
6	30–40 kDa	+	+	+	+	+	+	n.a	n.a
7	30–40 kDa	+	+	+	+	+	+	n.a	n.a

Scale: –, negative; ± , weakly positive; + , positive; n. a., not available.

The membranes of Fig. 1A–D were then incubated with stripping buffer to remove the primary and secondary antibodies and chromogenic substrate. The images confirmed that almost all PrP signals were diminished (Suppl. Fig. S3A–D, multiplex exposure: Suppl. Fig. S4). Next, another PrP antibody (EP1802Y) was used to re-examine the PrP signal patterns in these membranes, and the ~ 16–20 kDa and ~ 30–40 kDa smear bands were observed with the 3F4 antibody were also present with the EP1802Y antibody (Suppl. Fig. S5A–D).

**Figure 4:**
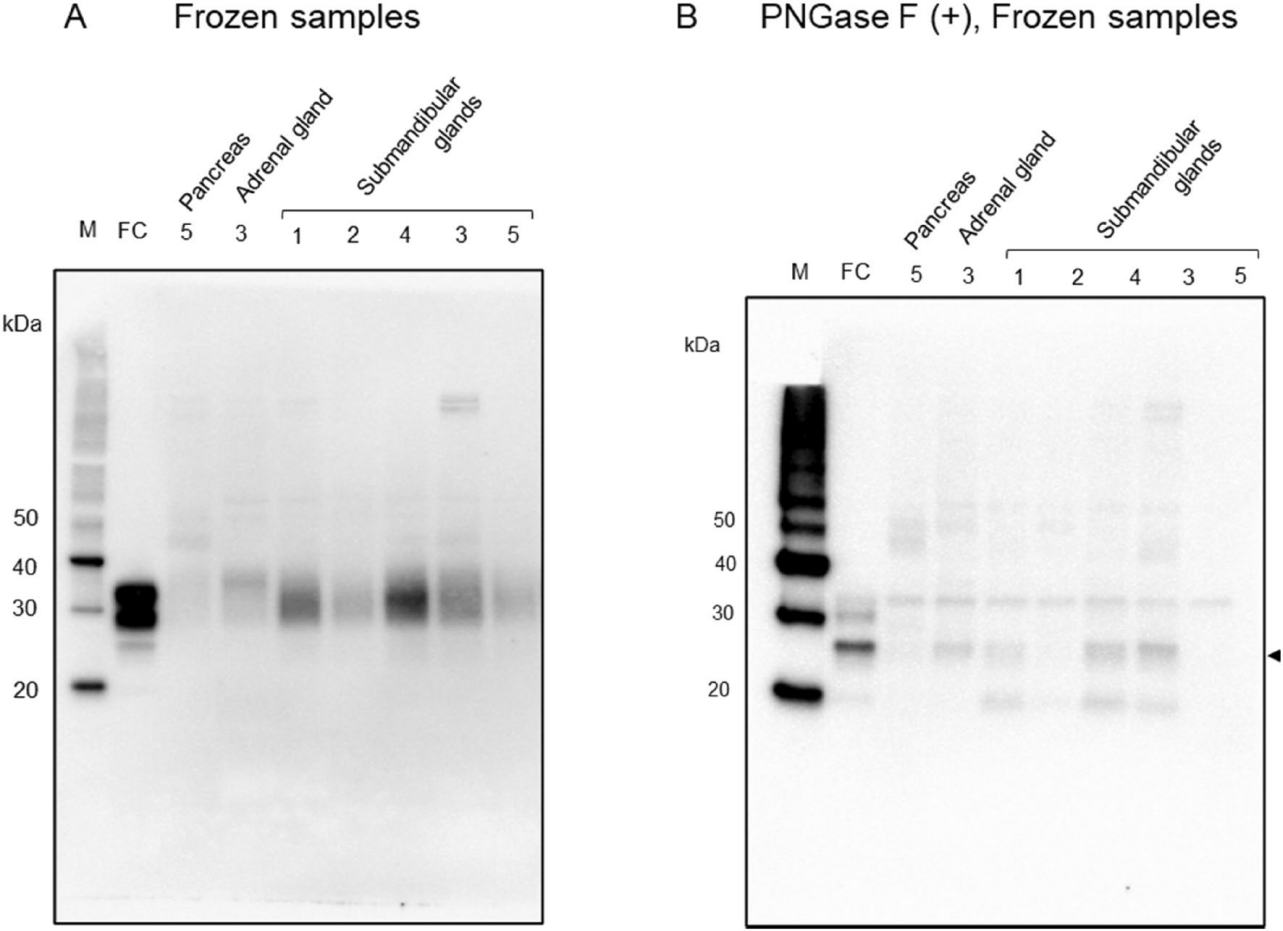
Western blot analyses from frozen samples (**A**) and western blot analysis after deglycosylated treatment (**B**). (**A**) In the frontal cortex (FC), PrP signals, nonglycosylated, strong mono and deglycosylated bands are observed at the molecular weight of 25–38 kDa. In the pancreas (case 5), adrenal gland (case 3) and submandibular glands (case 1–5), ~ 30–40 kDa PrP smear signals were noted. (**B**) After deglycosylated treatment with PNGaseF, 25 kDa PrP signals were most prominent in the frontal cortex, adrenal gland (case 3) and submandibular glands (case 1, 3 and 4).

Western blot analysis using frozen samples, 3F4 antibody showed unglycosylated, monoglycosylated, and diglycosylated PrP signals, ranging from 25 to 38 kDa in the frontal cortex (Fig. 4A). In the pancreas and adrenal gland, weak smear PrP signals were observed in 30–40 kDa. In the submandibular glands, although the densities were different depending on the cases, the smear PrP signals were also found in 30–40 kDa. Full length membrane of Fig. 4A is shown in the Suppl. Fig. S7. The − 16–20 kDa signals seen in the FFPE samples were not observed in the frozen samples. After deglycosylated treatment using PNGaseF, almost all samples showed a strong signal at the molecular weight of 25 kDa, which corresponds to the unglycosylated PrP molecular weight (Fig. 4B). Multiple exposure images of Fig. 4B are shown in Suppl. Fig. S8.

### Histopathological findings

**Figure 5:**
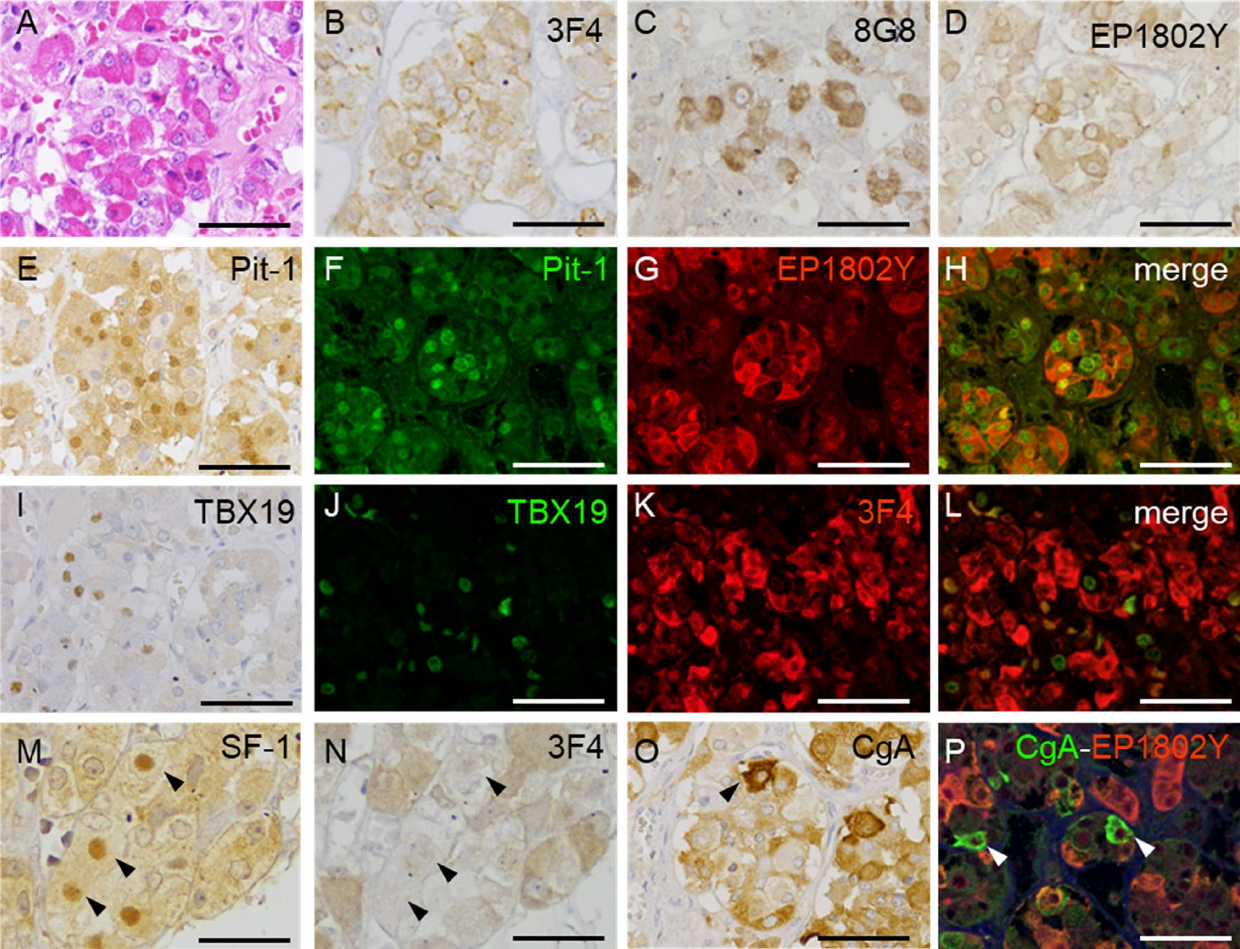
Cellular distribution of prion proteins (PrP) in the pituitary gland. (**A**) Hematoxylin and eosin staining show normal adenohypophyseal cell appearance (case 6). (**B**–**D**) Immunohistochemistry for PrP antibodies shows cytoplasmic PrP in adenohypophyseal cells (case 6; **B**: 3F4, **C**: 8G8, **D**: EP1802Y). (**E**) Immunohistochemistry for positive transcription factor 1 (Pit-1) reveals nuclear Pit-1. (**F**–**H**) Double immunofluorescence of Pit-1 and PrP (EP1802Y) shows that most cytoplasmic PrP-positive adenohypophyseal cells are immunopositive for nuclear Pit-1. (**I**) Immunohistochemistry for T-Box transcription factor 19 (TBX19) shows some adenohypophyseal cells with positive nuclei. (**J**–**L**) Most cytoplasmic PrP-positive cells are negative for nuclear TBX19. (**M**, **N**) On mirror images of immunohistochemistry for steroidogenic factor 1 (SF-1) (**M**) and PrP (**N**), most of the cytoplasmic PrP cells were negative for nuclear SF-1 (asterisks). (**O**) Most adenohypophyseal cells were positive for cytoplasmic chromogranin A (CgA), and several had strong expression (arrowhead). (**P**) Double immunofluorescence for CgA and PrP (EP1802Y) indicated colocalization in most cells, but cytoplasmic PrP was negative in cells strongly expressing CgA.

The adenohypophyseal cells had a normal appearance in all cases (HE stain; Fig. 5A). However, the immunostaining intensity per tissue depended on the PrP antibody and case (Table 3). Immunohistochemistry for PrP using 3F4 (Fig. 5B) and other PrP antibodies (Fig. 5C: 8G8, 5D: EP1802Y) revealed cytoplasmic staining in several adenohypophyseal cells. Also, immunohistochemistry for Pit-1 revealed nuclear staining in many adenohypophyseal cells (Fig. 5E). Furthermore, double immunofluorescence of PrP (EP1802Y) and Pit-1 showed that cytoplasmic PrP-positive adenohypophyseal cells were immunopositive for nuclear Pit-1 (Fig. 5F–H). In addition, most cytoplasmic PrP-positive adenohypophyseal cells were immunopositive for nuclear Pit-1 (74.3%, 55/74).

**Table 3:** Immunopositivity after prion protein immunostaining.

Case No	Pituitary glands			Adrenal glands			Pancreas			Submandibular glands		
	3F4	8G8	EP1802Y	3F4	8G8	EP1802Y	3F4	8G8	EP1802Y	3F4	8G8	EP1802Y
1	3	3	3	1	1	2	0	0	1	0	0	1
2	3	3	3	3	2	3	0	0	2	2	1	3
3	n.a	n.a	n.a	1	1	1	1	1	1	1	2	3
4	3	3	3	2	2	2	1	1	2	1	2	3
5	1	2	3	1	2	2	1	1	2	1	2	3
6	3	3	3	3	1	2	1	0	1	n.a	n.a	n.a
7	3	3	3	1	2	3	0	0	1	n.a	n.a	n.a

Scale: 0, negative; 1, mild; 2, moderate; 3, strong; n.a., not available. 3F4, 8G8, and EP1802Y: prion protein antibodies.

Immunohistochemistry for TBX19 revealed nuclear immunopositivity in some adenohypophyseal cells (Fig. 5I). Double immunofluorescence of PrP (3F4) and TBX19 (Fig. 5J–L) showed only a small number of cytoplasmic PrP-positive adenohypophyseal cells with nuclear TBX19 positivity (9.6%, 5/52). Immunohistochemistry for SF-1 revealed nuclear staining in the adenohypophyseal cells (Fig. 5M, arrowheads). However, double immunofluorescence of SF-1 and EP1802Y was difficult because of the different antigen activation methods. Therefore, we used a mirror image in the adjacent section. In the mirror image for SF-1 and PrP (3F4), most nuclear SF-1 positive adenohypophyseal cells were negative for cytoplasmic PrP (Fig. 5N, arrowheads). CgA immunopositivity was observed in most adenohypophyseal cells and was strongly expressed in some (Fig. 5O, arrowhead). Furthermore, double immunofluorescence of CgA and PrP (EP1802Y) revealed colocalization in most adenohypophyseal cells. However, highly CgA-expressing cells showed no cytoplasmic PrP (Fig. 5P, arrowhead).

**Figure 6:**
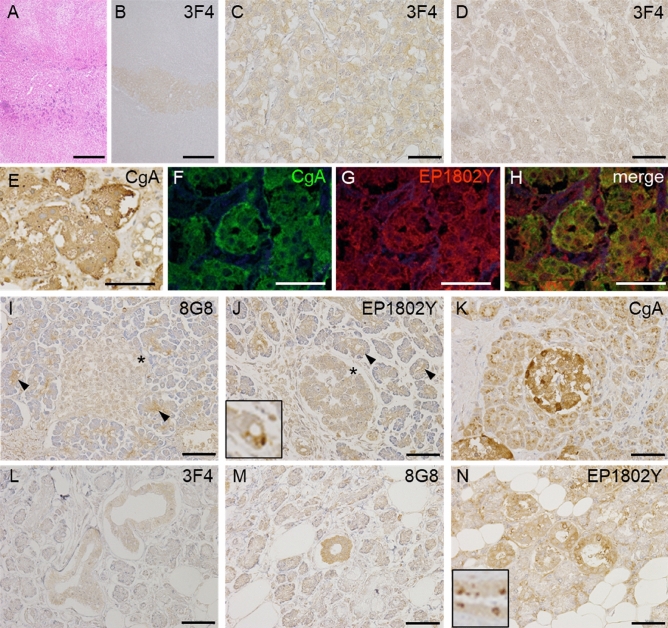
Histological and immunohistochemical findings in the endocrine and exocrine tissues (**A**–**H**: adrenal gland, **I**–**K**: pancreas, **L**–**N**: submandibular gland). (**A**) Hematoxylin and eosin staining show a normal appearance of the adrenal gland (case 6). (**B**, **C**) Immunohistochemistry for prion proteins (PrPs) (3F4) shows PrP immunopositivity in the adrenal medulla. (**D**) PrP immunopositivity (3F4) is very weak in the zona reticularis of the adrenal gland. (**E**) Immunohistochemistry for chromogranin A (CgA) shows CgA immunopositivity in the adrenal medulla. (**F**–**H**) Double immunofluorescence of CgA and PrP (EP1802Y) shows colocalization of CgA and PrP in the adrenal medulla. (**I**, **J**) Immunohistochemistry for PrP (**I**: 8G8, **J**: EP1802Y) shows weak immunopositivity in the islets (asterisks) and ductal epithelial (arrowheads) cells. Some ductal epithelial cells are strongly immunopositive for PrP (EP1802Y) (**J**: inset). (**K**) Islets cells are strongly immunopositive for CgA. (**L**, **M**, **N**) Immunohistochemistry for PrP (**L**: 3F4, **M**: 8G8, **N**: EP1802Y) shows immunopositivity in the ductal epithelia. Some ductal epithelial cells are strongly immunopositive for PrP (EP1802Y) (**N**: inset).

HE staining indicated a normal appearance in the adrenal gland (Fig. 6A), and immunohistochemistry for PrP (3F4) revealed marked PrP immunopositivity in the adrenal medulla (Fig. 6B,C). In contrast, PrP immunoreactivity was faint in the zona reticularis of the adrenal gland (Fig. 6D). Immunohistochemistry for CgA revealed marked immunopositivity in the adrenal medulla (Fig. 6E), and double immunofluorescence of PrP (EP1802Y) and CgA showed substantial colocalization of PrP and CgA (Fig. 6F–H). In the pancreas, islet cells had weak cytoplasmic PrP immunostaining (Fig. 6I,J, asterisks). However, the ductal epithelia were also positive for PrP (Fig. 6I: 8G8, arrowheads; Fig. 6J: EP1802Y, arrowheads), and some ductal epithelia showed marked PrP staining (Fig. 6J, inset). Immunohistochemistry for CgA revealed marked immunopositivity in the pancreatic islet cells (Fig. 6K). In the submandibular gland, most ductal epithelia were PrP-positive (Fig. 6L: 3F4, 6M: 8G8, 6N: EP1802Y). The ductal epithelia showed various immunopositivity intensities depending on the antibody: 8G8 and EP1802Y were strong, but 3F4 was mild. In addition, some ductal epithelia showed marked PrP staining (Fig. 6N, inset).

## Discussion

This study demonstrated the characteristic molecular properties of PrP in several human endocrine and exocrine tissues, such as the pituitary, adrenal, and submandibular glands and the pancreas, which have different characteristics than the brain. Additionally, we detail the histological localization of PrP in these tissues.

Western blotting showed PrP smear bands ranging from 30 to 40 kDa in the pituitary gland, similar to previous reports^5^. These molecular weights are higher than those for the brain, and our previous study showed that this was due to excessive glycosylation. In western blot analysis from FFPE samples, PrP signals were observed in the adrenal, pancreas, and submandibular glands at ~ 30–40 kDa and ~ 16–20 kDa. However, western blot analysis from frozen samples showed no ~ 16–20 kDa signals. Therefore, the ~ 16–20 kDa signals seen in FFPE samples were likely to be an artifact that occurred in the fixation and embedding procedures the FFPE samples. The ~ 30–40 kDa PrP smear signal was similar to that of the pituitary gland. Both in the FFPE samples and frozen samples, after deglycosylation treatment with PNGaseF, the ~ 30–40 kDa PrP smear signal was converged to a single band at the molecular weight of about 25 kDa which corresponds to a nonglycosylated PrP signal. Therefore, PrP of organs such as the pancreas, adrenal gland, and submandibular gland also undergoes excessive glycosylation compared to the cerebrum. Unlike PrP in the cerebrum, PrP in endocrine/exocrine tissues may be difficult to separate by excessive glycosylation like pituitary PrP.

Pit-1 activates transcription of the GH, prolactin, and beta-thyroid stimulating hormone genes^19^, and we found that most cytoplasmic PrP^c^ positive adenohypophyseal cells were immunopositive for nuclear Pit-1. Conversely, cytoplasmic PrP^c^ positive adenohypophyseal cells were essentially negative for SF-1 and TBX19. These findings suggest that the expression of PrP^c^ in adenohypophyseal cells tents to occur in differentiated cells downstream from somatotroph stem cells rather than corticotroph cells and gonadotroph cells. A previous report showed that cytoplasmic PrP^c^ positive adenohypophyseal cells are frequently positive for GH or prolactin^5^, and our results agree. However, there are no reports that suggest a direct association between PrP and Pit-1, and it is unclear when PrP^c^ is highly expressed during cell differentiation by Pit-1.

In the adrenal gland, PrP^c^ immunoreactivity was observed in the adrenal medulla. The adrenal medulla is derived from the neural crest, and PrP^c^ is as abundant as in the central nervous system. For example, PrP^Sc^ was detected in the adrenal medulla in cattle affected with bovine spongiform encephalopathy and scrapie-infected sheep^20,21^. In humans, we previously reported PrP^Sc^ aggregation in the adrenal medulla of sCJD patients and enhanced seeding activity on real-time quaking-induced conversion^22^. Our results suggested that PrP^c^ in the adrenal medulla may be the source of PrP^Sc^.

In the pancreas, islet cells have cytoplasmic PrP. In rats, PrP^c^ is abundant in islet cells and is involved in regulating blood glucose homeostasis^23^. In addition, PrP^c^ primarily expresses in pancreatic β cells in humans and may contribute to insulin resistance^24,25^. CgA is a neuroendocrine protein that is found in the adrenal medulla and sympathetic nerve endings and is localized to nerve cells and secretory vesicles of endocrine cells^26^. CgA function as a low-affinity Ca2+ binding protein in secretory granules of neuroendocrine cells. In addition, CgA are also observed in the lumen of the ER of many cell types and play modulatory role in the control of Ca2+ release from ER. CgA is a precursor of bioactive peptide including pancreastatin, vasostatin, chromostatin, catestatin and parastatin. Reports have shown that PrP^c^ and CgA colocalized in bovine islet cells.^27^ In our study, CgA and PrP^c^ colocalization was noted in adenohypophyseal cells, pancreatic islet cells, and the adrenal medulla, suggesting that PrP^c^-expression in neuroendocrine cells could be expressed throughout the body of human. However, very high levels of CgA-expressing adenohypophyseal cells did not show cytoplasmic PrP. Therefore, even if the genes for CgA and PrP^c^ are adjacent to each other, there is a difference in function, they do not necessarily colocalize in all tissues. The details of the relationship between CgA and PrP have not yet been clarified, and future studies are important.

PrP immunopositivity was detected in the ductal epithelium of the submandibular gland and the pancreas. There have been no reports of PrP expression in the ductal epithelium of animals or humans. CWD is a fatal neurodegenerative prion disease that affects species of the Cervidae family and is now a widespread global problem in the United States, Canada, and Europe^12,28,29^. Saliva and feces containing PrP^Sc^ are likely sources of infection^28,29^. Our results suggest that in CWD animals, PrP^Sc^ may be secreted from the ductal epithelium in saliva and from the pancreas through the gastrointestinal tract to feces.

This study detected PrP^c^ in human endocrine and exocrine tissues using western blotting and clarified the histological details. PrP^c^ is an element of the crinophagy mechanism (an autophagy mechanism that processes excess hormones and neurotransmitters) in secretory cells. Furthermore, PrP^c^ is involved in the secretory pathway of secretory granules^8^. The immunohistochemistry and western blotting detection sensitivities for PrP^c^ varied between cases. This may be due to different endocrine and exocrine function states per case during the period before death. The immunohistochemistry detection sensitivities also differed depending on the PrP antibody, which may be related to antigen exposure owing to the three-dimensional structure of PrP^c^. This study includes several limitations. Frozen samples of endocrine and exocrine tissue were not sufficiently collected at autopsy, therefore, analysis of western blots was mainly performed from FFPE samples. It is undeniable that modification of the sample during the FFPE manufacturing process can affect the results of western blot. However, western blot results from FFPE of the brain and pituitary gland were similar to those of frozen samples. In addition, since it is a biochemical result of the tissue adjacent to the specimen used for histological observation, we think that it is also a very suitable method for examining the tissue localization of PrP^c^. Therefore, we believe that the characteristic signals found in western blots of the adrenal glands, pancreas and submandibular glands are reliable.

## Conclusion

In this study, we demonstrated PrP^c^ in human endocrine/exocrine tissues including pituitary, adrenal, submandibular glands and the pancreas. Recently, in prion diseases, PrP^Sc^ deposits have been observed not only in the central nervous system but also in systemic organs including endocrine tissues. Our results may indicate a source of PrP^Sc^ in systemic organs. At the same time, they can also be a source of infection. The detailed function of PrP^c^ in endocrine and exocrine tissues is still unknown. Therefore, clarifying the relationship between PrP^c^ and secretory function, and monitoring endocrine and exocrine function in prion disease patients are important for infection control and prompt diagnosis.

## Supplementary Information

Supplementary Information.

## Data Availability

All data supporting the findings of this study are available within the article and its supplementary files.
