# Supplementary material for: Characteristic distribution and molecular properties of normal cellular prion protein in human endocrine and exocrine tissues
Source: Sci Rep. 2022 Sep 10;12:15289. doi: 10.1038/s41598-022-19632-4 (PMC9464206; doi:10.1038/s41598-022-19632-4)
Supplement: Supplementary file 1 — Supplementary Information. [file 41598_2022_19632_MOESM1_ESM.pdf]

Supplementary Figure 1: Full length membranes of Figure 1

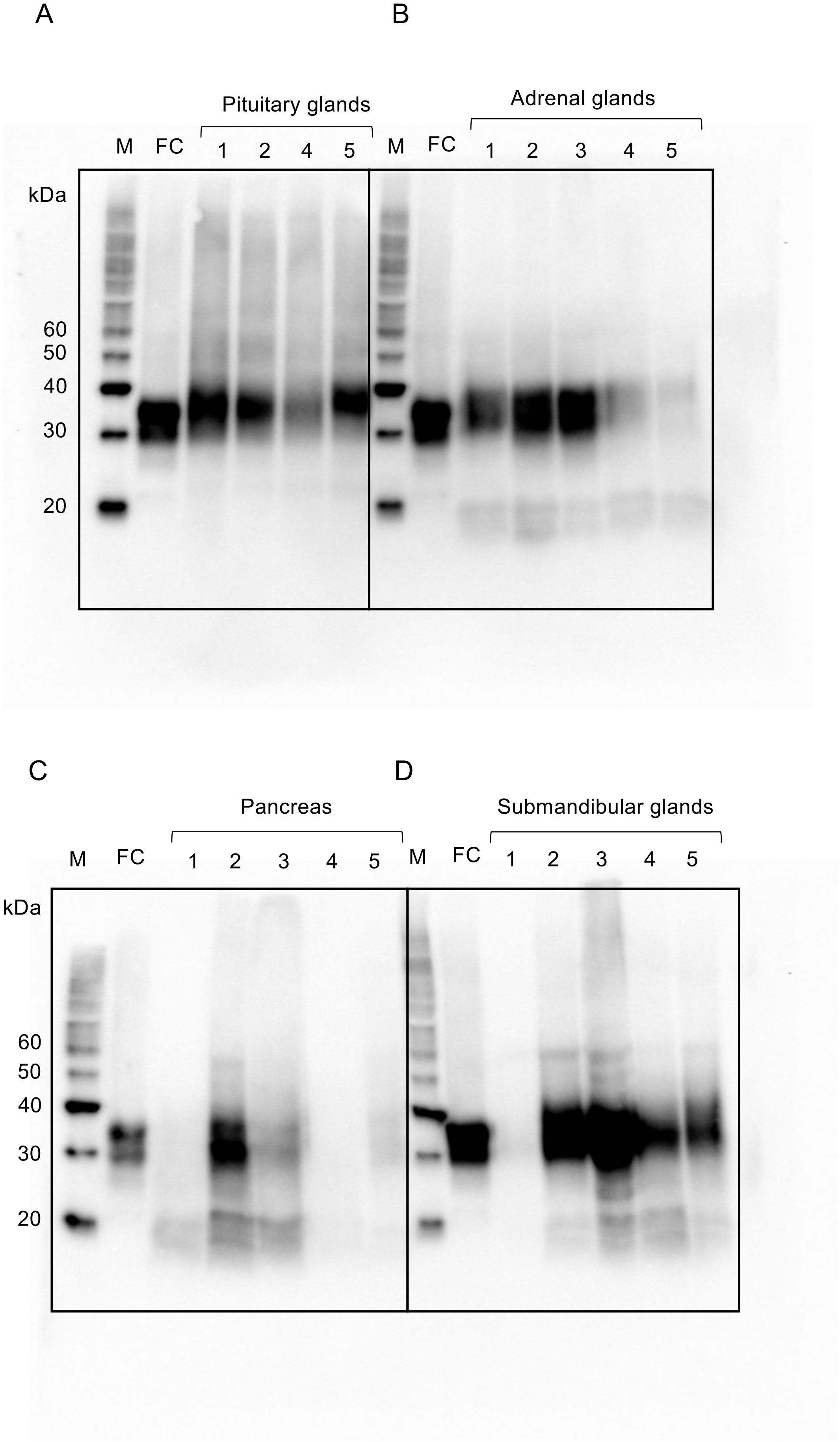

Supplementary Figure 1. Full length membranes of Figure 1 (ABCD) are shown.

Supplementary Figure 2: Full length membranes of Figure 2

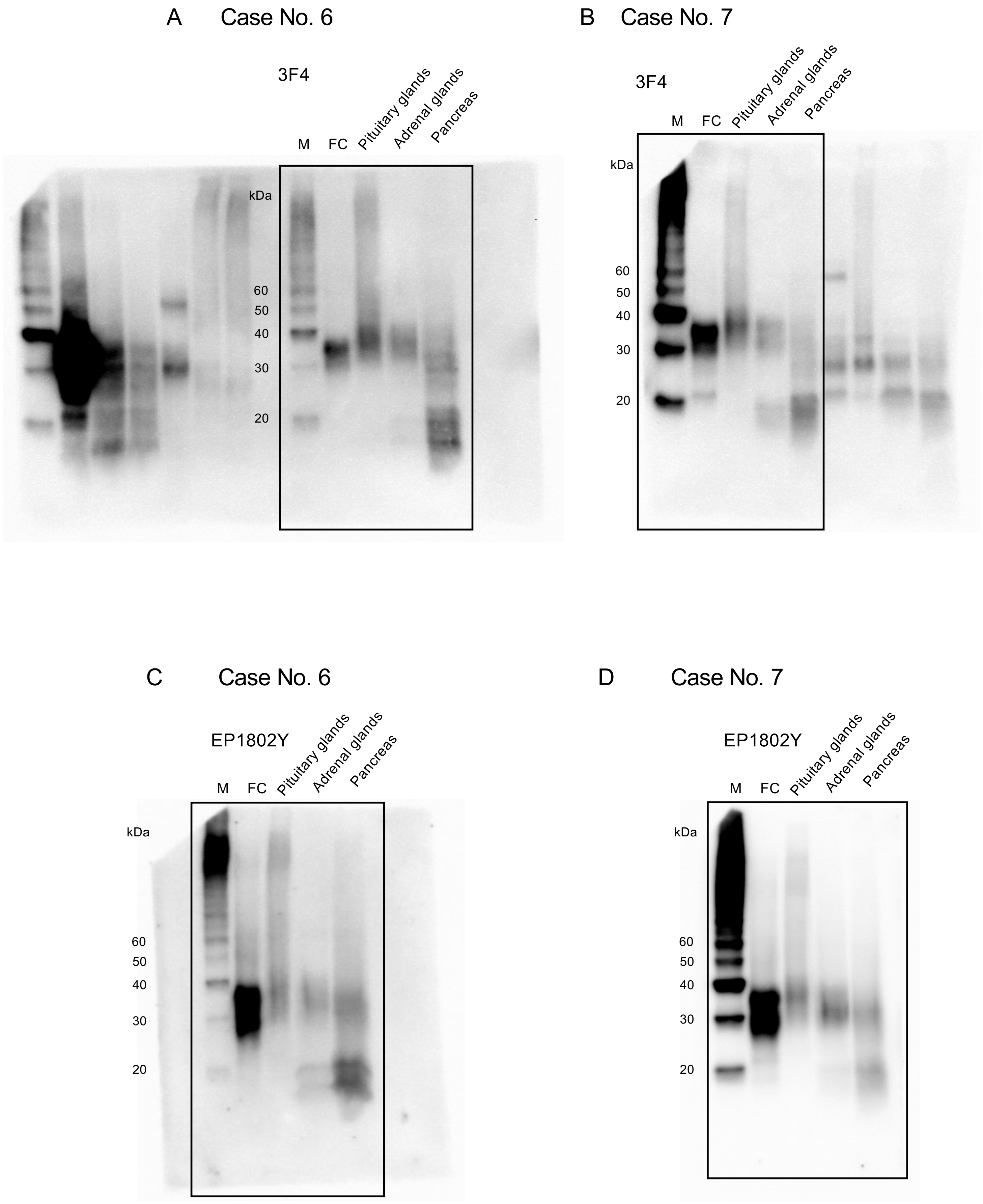

Supplementary Figure 2. Full length membranes of Figure 2 (ABCD) are shown.

Supplementary Figure 3: After stripping of Fig. 1

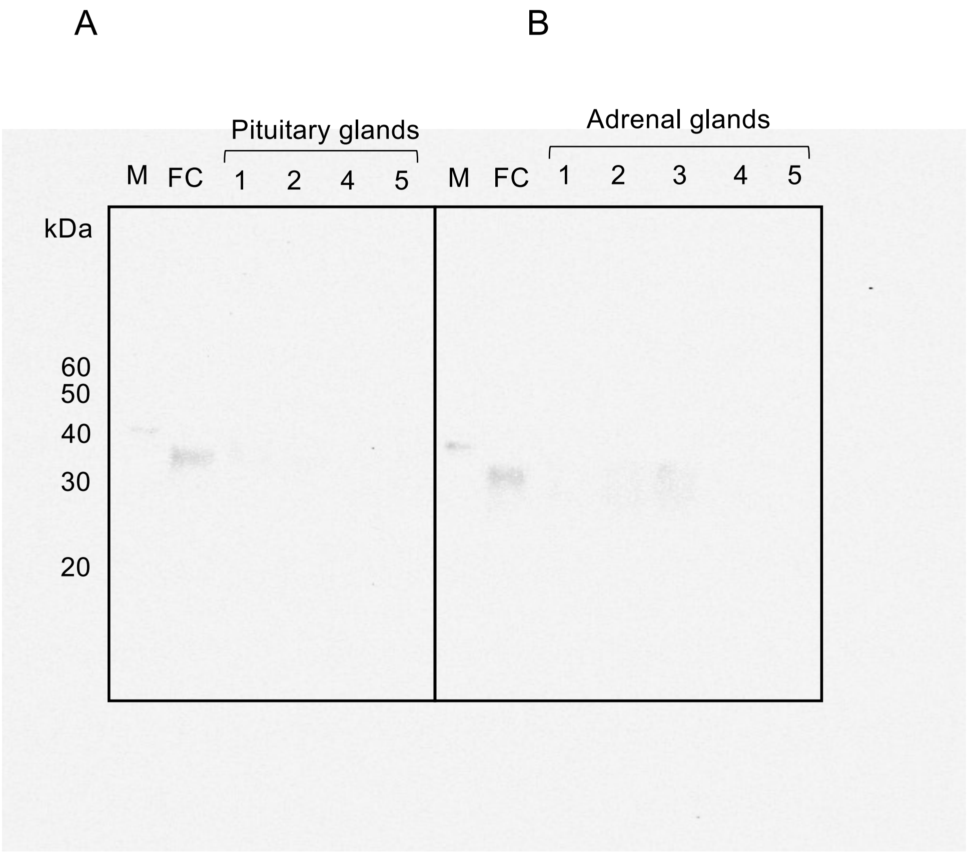

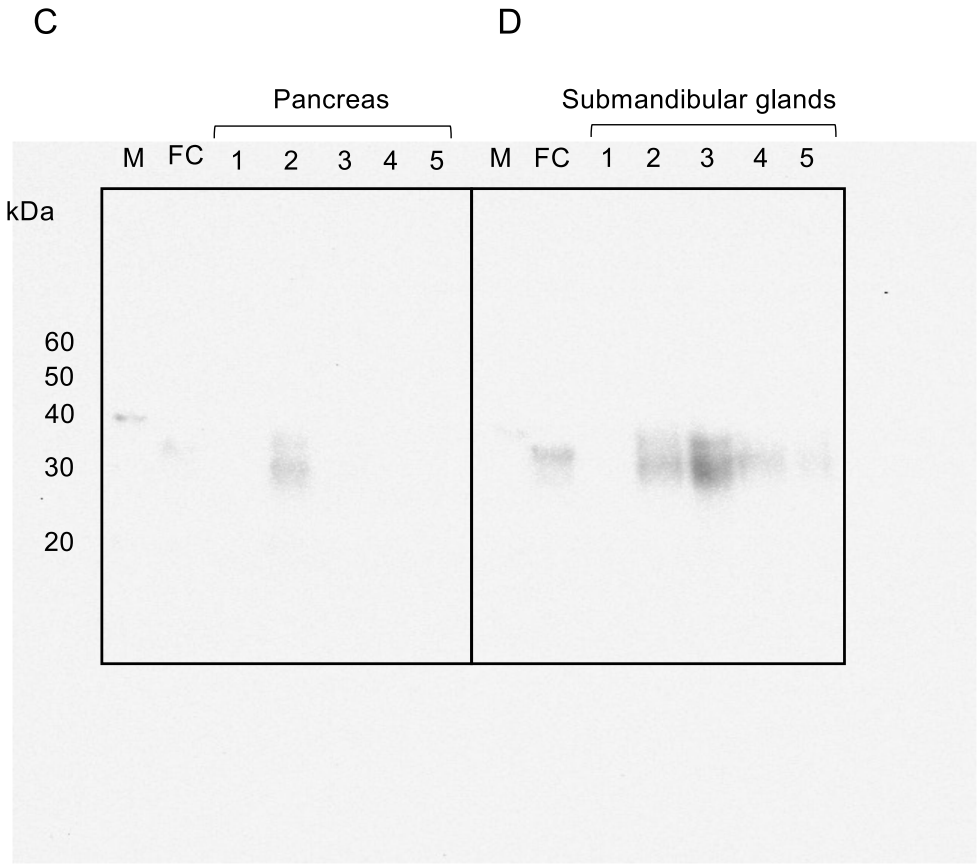

Supplementary Figure 3. The primary and secondary antibodies and chromogenic substrate were removed by incubating with stripping buffer. (A, B) Almost all 3F4 signals were unidentifiable. However, a very weak prion protein signal remained in the (C) case 2 pancreas sample and (D) case 2 and 3 submandibular gland samples.

Supplementary Figure 4: Multiplex exposure images of Supp Fig. 3

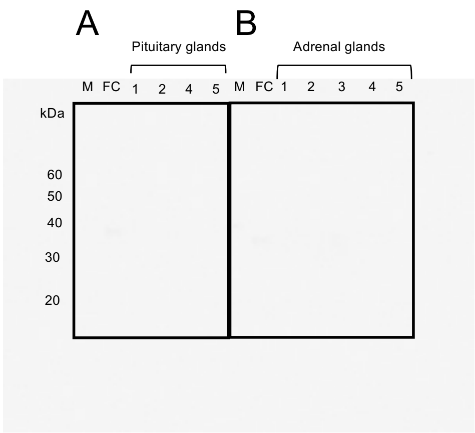

Exposure time: 1 min

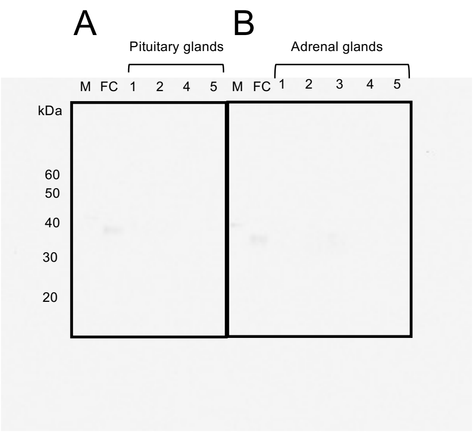

Exposure time: 3 min

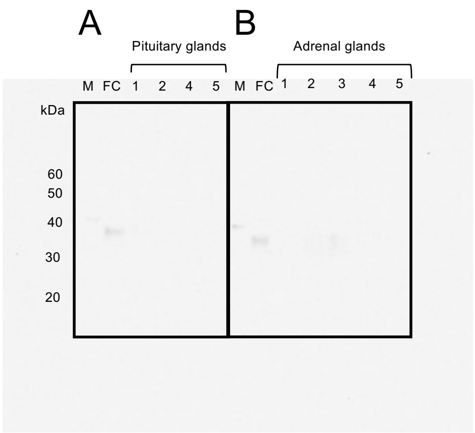

Exposure time: 5 min

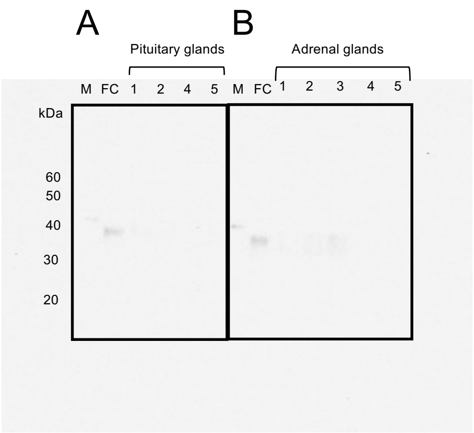

Exposure time: 7min

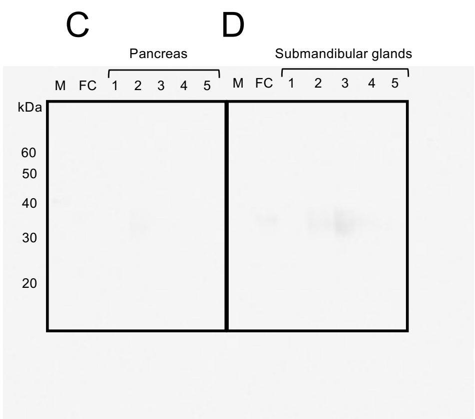

Exposure time: 1 min

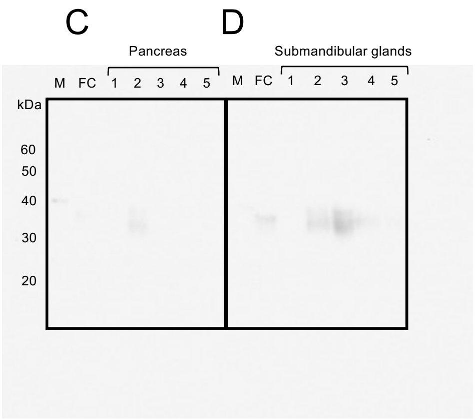

Exposure time: 3 min

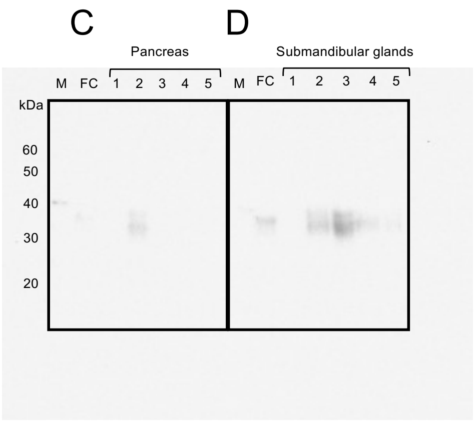

Exposure time: 5 min

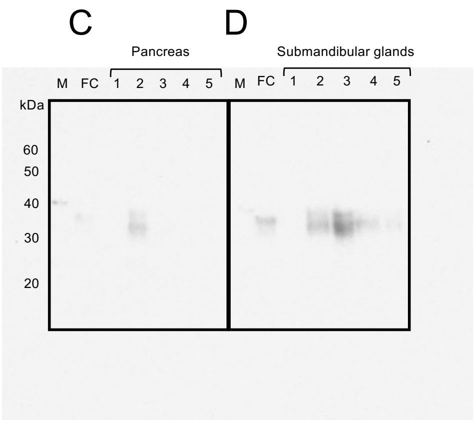

Exposure time: 7 min

Supplementary Figure 4. Multiplex exposure images of Supp. Fig. 3 (ABCD) are shown.

Supplementary Figure 5: Western blot analyses using EP1802Y antibody after stripping treatment

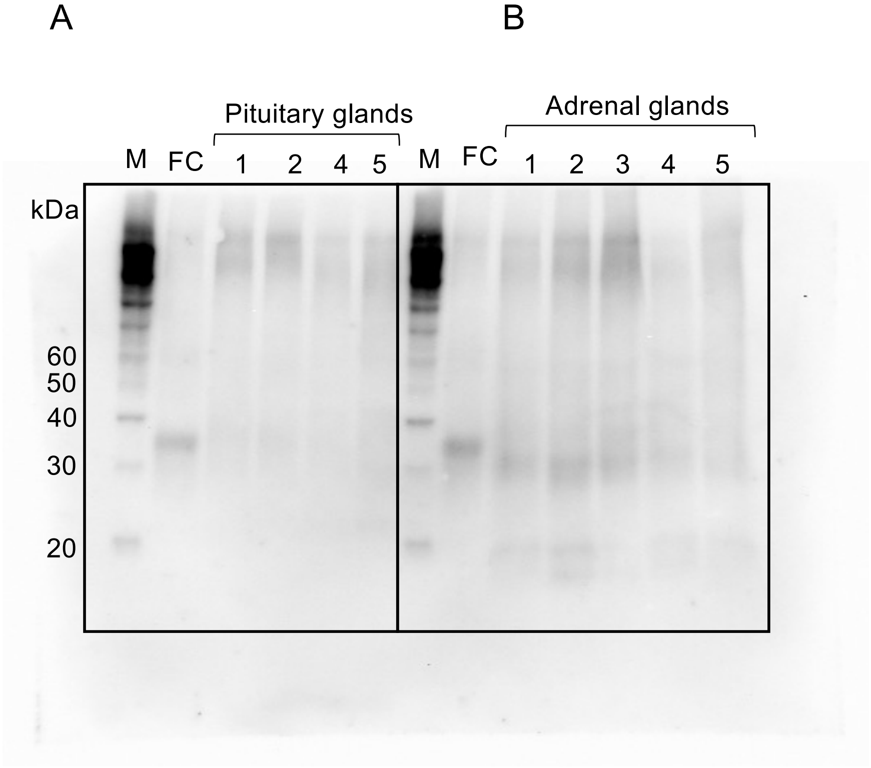

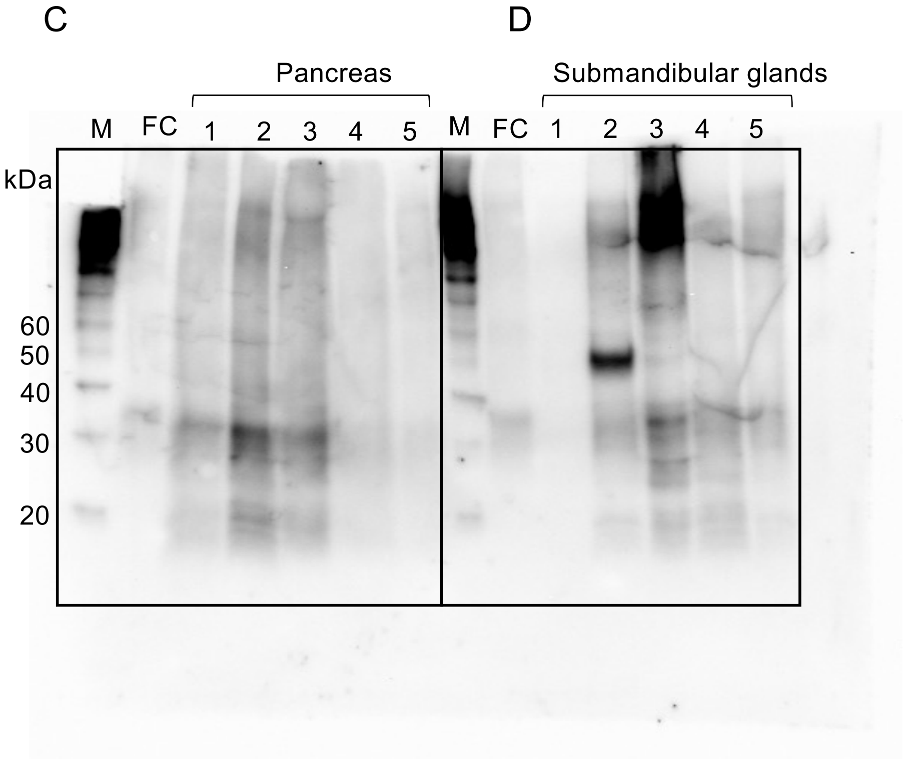

Supplementary Figure 5. Western blot analyses with the EP1802Y anti-prion protein (PrP) antibody. (A) The frontal cortex (FC) samples show ~25–38 kDa PrP signals, and the pituitary gland samples show weak PrP signals of ~30–40 kDa (cases 1, 2, 4, and 5). (B) The adrenal gland samples (cases 1–5) show ~30–40 kDa and ~16–20 kDa PrP signals. (C) The pancreas samples (cases 1–5) show ~30–40 kDa and ~16–20 kDa PrP signals (cases 1–3). The PrP signal was difficult to identify in cases 4 and 5. (D) The submandibular gland samples (cases 1–5) show ~30–40 kDa and ~16–20 kDa PrP signals (cases 2–5). The PrP signal was difficult to identify in case 1.

Supplementary Figure 6 : Full length membranes of Figure 3E: Case No. 7

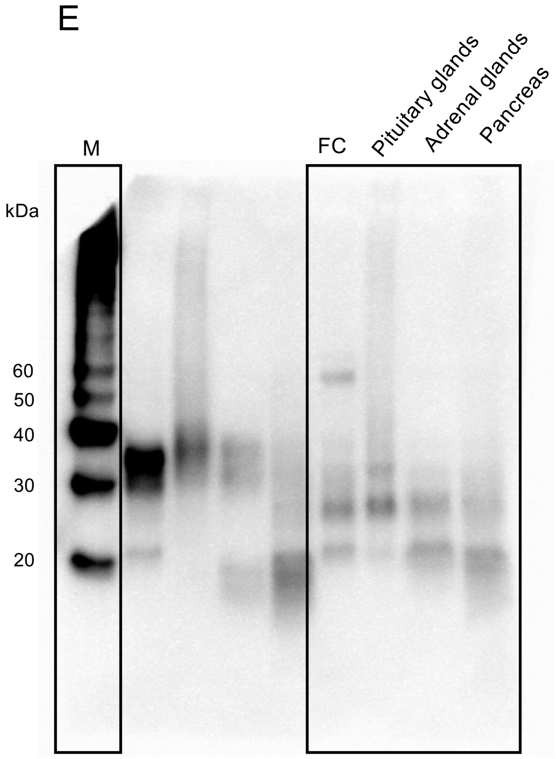

Supplementary Figure 6. Full length membrane of Fig. 3E (case 7) is shown.

Supplementary Figure 7 : Full length membranes of Figure 4A

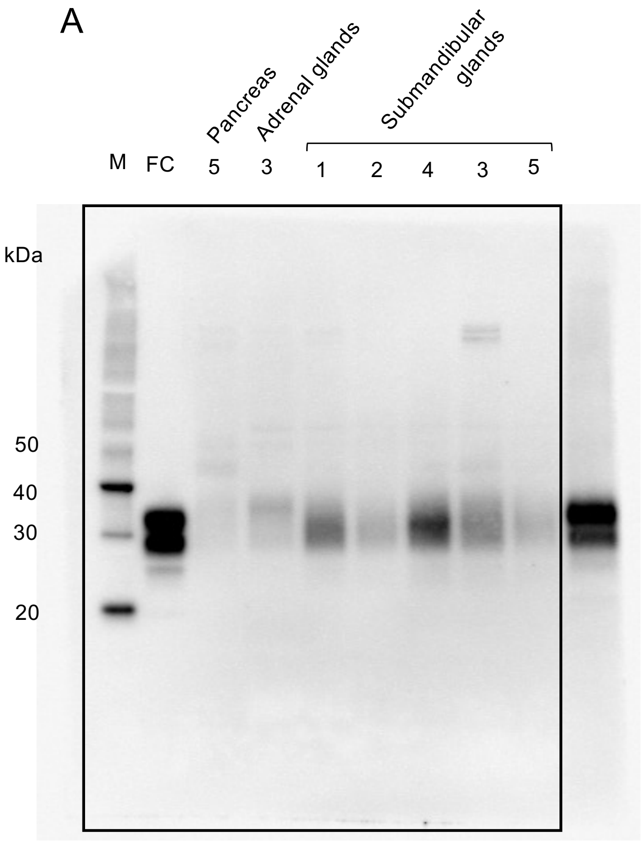

Supplementary Figure 7. Full length membrane of Fig. 4A is shown.

Supplementary Figure 8 : Multiplex exposure images of Fig. 4B

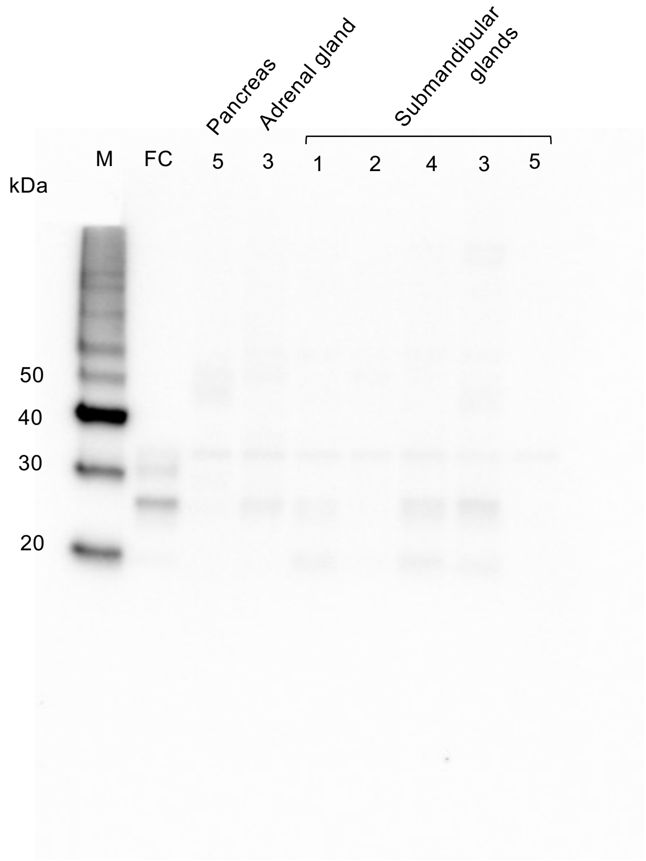

Exposure time: 5 min

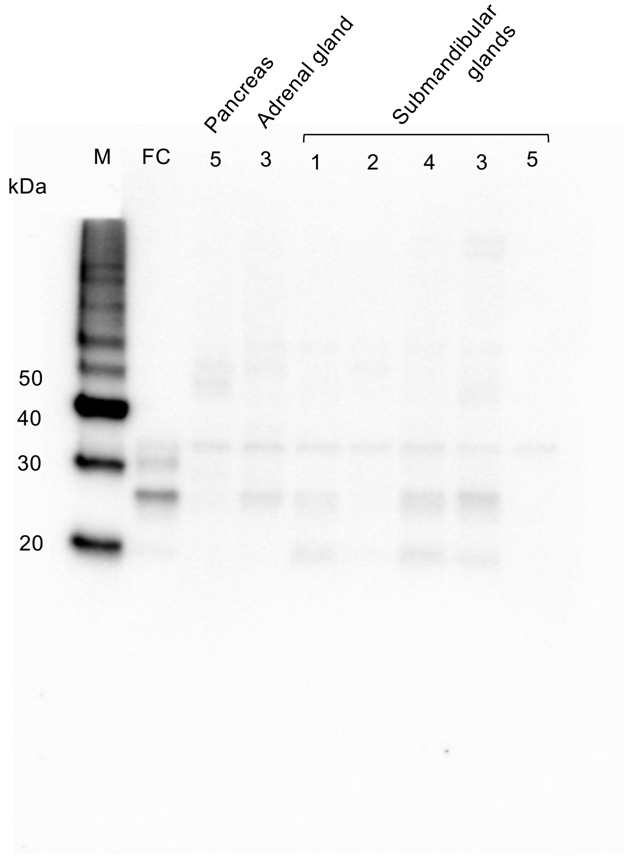

Exposure time: 10 min

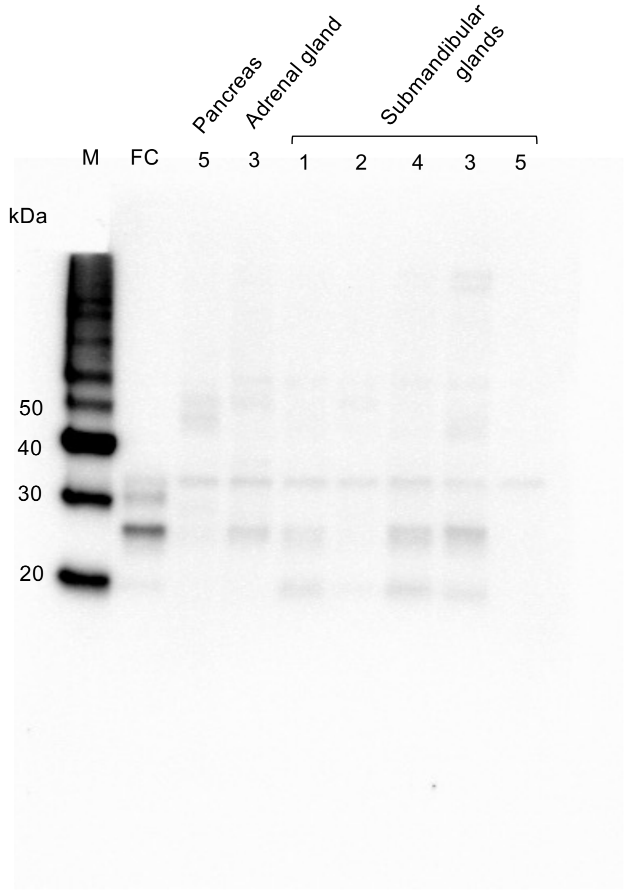

Exposure time: 15 min

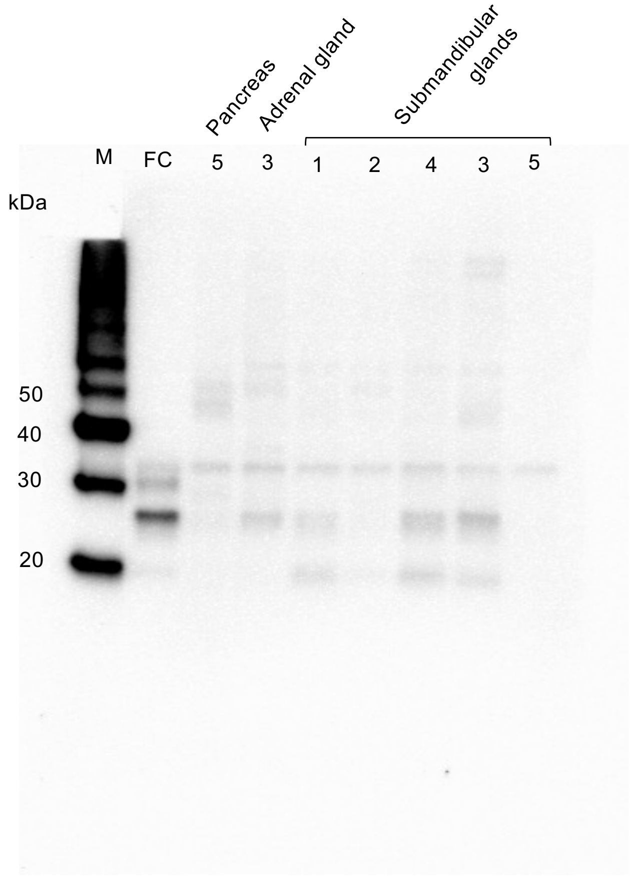

Exposure time: 20min

Supplementary Figure 8. Multiplex exposure images of Fig. 4B are shown.
